# Recreation in Relation to the Health of the People

**Published:** 1891-01-24

**Authors:** 


### Recreation in Relation to the Health of the People.

### I.—INTRODUCTORY.

What is perfect health? Is it not a state in which not merely the physical, but the mental and moral powers of a man have free development, space and opportunity being given, not for morbid growth, but for a natural and a fairly proportionate exercise of them all? We say "fairly" proportionate, because it is evident that, as things go, and indeed as human beings are made, it is impossible to develop to the full all the various powers of any one man; the brief span of his existence itself would not permit of this, much less the exigencies of life. But one thing is clear; that in a well-ordered community every man, woman, and child should have a chance of attaining to at least a moderate amount of what we may term all-round health; and it is indeed lamentable to reflect how far are many thousands of our fellow-creatures from securing that chance.

Of course there are numerous aspects under which this question of health may be considered. For the present, we choose out but one, and propose to discuss, in a short series of articles, the question of Recreation for the People,—that recreation which we believe is essential to their happiness and well-being—in a word, to their health. Not that we by any means intend to depreciate work. If work and recreation must be pitted against each other, who can doubt that work must win the day? As Mrs. Browning forcibly put it, "God says, sweat for foreheads"; and we are by no means sure that the unfortunate worker who slaves all day for his pittance of bread is not more to be envied than the moneyed idler, who never needs to be recreated, because he never knows what it is to spend his strength in his own or anybody else's service. Let us preach work to him by all means; but let us also preach recreation to the busy toiler, or rather to those who have it in their power, more or less, to procure for him what he might seek in vain to procure for himself.

True, the subject is by no means a new one. Much has been said of late, and, better still, much has been done, with the object of securing recreation for the masses; and it is, perhaps, the best proof of how deeply men's minds have been stirred on this subject, that an opposition party has begun to make itself heard, declaring that a new religion is being preached—the religion of amusement; that the rising generation is being taught to play instead of to work, and that when once an appetite for amusement is created, it will grow and spread to a disastrous extent. Well, we might answer to this that the appetite for amusement has not been created—it is as old as Adam, and that what is sought by preachers of recreation is to direct it into safe and harmless channels. But the fact that many sensible people are to be found on the opposition benches with regard to this question, makes it well worth our while to pause a few moments, and inquire how it is that they do not go with the stream.

For one thing, their quarrel will very often be found to be with recreation, not for the masses, but for the classes. And here, we do think, they have some ground for complaint. Games are excellent in their way, but they are not the main business of life; and when one hears that at certain schools the intellectual attainments of a candidate for a mastership are as nothing compared with his proficiency at cricket or "footer"—when one listens to the talk of both boys and masters, and finds youths at college declaring in all innocence and sincerity, when consulted by Paterfamilias as to the advisability of sending Bobby to a certain school—"You won't find a better anywhere, Pater. Why, they've licked all the cricket teams round!"—when one hears all this, we say, it is impossible not to feel that recreation takes too prominent a place in the thoughts and lives of the young in our upper classes. Work should be always put in the first place, play in the second; and in these days, when the professions are already overcrowded, and hard work is essential to success, it seems almost cruel to bring up our boys in habits and ideas so little applicable to their after-life.

"Of course the governor meant awfully well, but I wish he'd never sent me to college," was said to the writer by a young solicitor. "To have such an easy, jolly time of it, and then to come to this beastly grind! I don't know how to bear it sometimes."

But with the working classes, it is a very different matter. In their lives there is too often not only no provision for amusement of a healthful and harmless kind, but no chance of obtaining it; and if some among the upper classes have all play and no work, many more among the lower classes have all work and no play.

Another cause for the divergence of opinions on this question is, no doubt, to be found in the fact that old age and youth do not see with the same eyes. It is generally old or middle-aged persons who inveigh against amusement. They no longer feel the need for it, and cannot understand that youth craves and indeed will have it, in some form or another. An evening spent by their own quiet fireside is far more attractive than any outside entertainment could be; and, even as to books, it is by no means uncommon to find old or middle aged people, both in the lower and middle classes, who will tell you in all sincerity that they "never read anything but the Bible now—it's the best of all books, and to read a story-book seems a waste of time, somehow." Let such persons legislate for the young, and unless they have quick sympathies, and a vivid recollecton of feelings and desires long since passed away, they are sure to decide that recreation is not necessary, and to find out their mistake by bitter experience.

Some, again, declare, as has been said, that the duty of providing amusement for the people is exalted into a religion, and are confirmed in their fears by seeing how much time and thought are given to the matter by certain clergymen, to the injury (so it is said) of their proper work. It is not our business here to criticise the clergy, or to say what proportion of their time should be given to one duty, and what proportion to another; but we can quite believe it to be possible that some young clergymen, deeply impressed with the need of the people for more recreation, may have made the mistake of taking up work which might have been equally well done by laymen, and have yet been accorded the support and sanction of the Church. But it must not be forgotten that there are indirect as well as direct ways of caring for souls; and if a clergyman succeeds in impressing on his people that religion may and should enter into every department of life, he cannot be said to be working in vain. It is surely high time that the Church should repudiate, for good and all, the Puritanical and unnatural doctrine that amusements are "worldly," and that the true Christian should have nothing to do with them. To a great extent this has been already done; but the doctrine still lingers in certain places and in certain sets.

"I put it to you plainly," said a clergyman not long ago, to a country congregation. "Do you not feel, after going to an evening party, that you would have been much better at home, reading your Bible or saying your prayers?" Naturally, religion, as presented by him, wore a most unattractive aspect to the young. Life is not all prayers and Bible reading, any more than it is all beer and skittles; and, if the truth were known, the worthy gentleman himself was probably engaged in more secular occupations for at least part of the time during which his hearers were disporting themselves at the "evening party." Such teaching is, indeed, unreal as well as mischievous; and if it had not been for the Puritanism of our ancestors, there would probably have been many more converts to religion, while it is difficult to believe but what our national amusements would by this time have reached a higher level than now is theirs. Surely it is the world which needs regenerating, not a handful of people out of the world; and those who give up everything "worldly" lose the regenerative influence which they are meant to have upon society, and make the same mistake—with, indeed, far less excuse for it—that was made by the monks and nuns of former days.

All honour, then, to those teachers of religion who recognise that a love of amusement is part of the natural and the God-given constitution of man, and seek to put within his reach means by which he may innocently gratify it. They have doubtless yet higher work to do, which must claim the first and best of their attention; but no department of life, however small, should be treated as though it were outside the pale of religion. It was of smaller matters than these that the Pharisees were told: "These ought ye to have done, but not to leave the other undone."

